# Copper chelation with tetrathiomolybdate suppresses adjuvant-induced arthritis and inflammation-associated cachexia in rats

**DOI:** 10.1186/ar1801

**Published:** 2005-08-08

**Authors:** Atsushi Omoto, Yutaka Kawahito, Igor Prudovsky, Yasunori Tubouchi, Mizuho Kimura, Hidetaka Ishino, Makoto Wada, Makie Yoshida, Masataka Kohno, Rikio Yoshimura, Toshikazu Yoshikawa, Hajime Sano

**Affiliations:** 1 Inflammation and Immunology, Graduate School of Medical Science, Kyoto Prefectural University of Medicine, Kyoto, Japan; 2 Center for Molecular Medicine, Maine Medical Center Research Institute, Scarborough, Maine, USA; 3 Department of Urology, Osaka City Graduate School of Medicine, Osaka, Japan; 4 Division of Rheumatology and Clinical Immunology, Department of Internal Medicine, Hyogo College of Medicine, Nishinomiya, Japan

## Abstract

Tetrathiomolybdate (TM), a drug developed for Wilson's disease, produces an anti-angiogenic and anti-inflammatory effect by reducing systemic copper levels. TM therapy has proved effective in inhibiting the growth of tumors in animal tumor models and in cancer patients. We have hypothesized that TM may be used for the therapy of rheumatoid arthritis and have examined the efficacy of TM on adjuvant-induced arthritis in the rat, which is a model of acute inflammatory arthritis and inflammatory cachexia. TM delayed the onset of and suppressed the severity of clinical arthritis on both paw volume and the arthritis score. Histological examination demonstrated that TM significantly reduces the synovial hyperplasia and inflammatory cell invasion in joint tissues. Interestingly, TM can inhibit the expression of vascular endothelial growth factor in serum synovial tissues, especially in endothelial cells and macrophages. Moreover, the extent of pannus formation, which leads to bone destruction, is correlated with the content of vascular endothelial growth factor in the serum. There was no mortality in TM-treated rat abnormalities. TM also suppressed inflammatory cachexia. We suggest that copper deficiency induced by TM is a potent approach both to inhibit the progression of rheumatoid arthritis with minimal adverse effects and to improve the well-being of rheumatoid arthritis patients.

## Introduction

Rheumatoid arthritis (RA) is a chronic, destructive inflammatory polyarticular joint disease. It is characterized by massive synovial proliferation and subintimal infiltration of inflammatory cells, which along with angiogenesis leads to the formation of a very aggressive tissue called pannus [1,2]. Expansion of the pannus induces bone erosion and cartilage thinning, leading to the loss of joint function. The rheumatoid pannus can thus be considered a local tumor. One of the earliest phenomena observed in RA is synovial neovascular formation delivering nutrients and oxygen to this proliferating pannus [3]. It has been demonstrated that angiogenesis inhibitors can inhibit the growth of pannus in animal arthritis models [4]. Vascular endothelial growth factor (VEGF) plays a pivotal role in the pathogenesis of RA [3,5,6]. Immunohistochemical and *in situ *hybridization studies indicate that VEGF is strongly expressed in subsynovial macrophages, in fibroblasts surrounding microvessels, in vascular smooth muscle cells, and in synoviocytes [7-9]. VEGF expression is activated at the very early stages of RA, and it continues throughout the course of the disease [10,11]. The VEGF level in synovial fluid and tissues correlates with the clinical severity of RA and with the degree of joint destruction [10]. Moreover, VEGF mediates the recruitment, chemotaxis, and proliferation of osteoclast precursor macrophages, leading to bone destruction [11,12].

RA is also characterized by increased production of the inflammatory cytokines tumor necrosis factor alpha (TNF-α) [1], IL-1α [13], IL-1β [1], and fibroblast growth factor (FGF) 1 [14]. TNF-α appears to be a key mediator in the disease process, and IL-1β plays a permissive role by acting to shift the whole-body protein metabolism towards net catabolism, to elevate resting energy expenditure, and to increase joint pain and stiffness [15]. Treatment with antibodies against TNF-α, IL-1α, and IL-1β attenuated RA in the experimental mouse model [16]. FGF-1 is important for the growth of synoviocytes in the course of RA [17].

Rheumatoid cachexia was first described more than a century ago [18]. However, it has not been recognized as a common problem among patients with RA until relatively recently. Rheumatoid cachexia leads to muscle weakness, osteoporosis, and a loss of functional capacity. It also increases susceptibility to infection [19], and is believed to accelerate morbidity and mortality in RA [15].

Copper is an essential trace element that acts as a cofactor for a variety of enzymes by virtue of its ability to accept and donate electrons under physiologic conditions [20]. Additionally, copper ions have recently been demonstrated to be required for the assembly of multiprotein release complexes in the process of stress-induced nonclassical release of FGF-1 and IL-1α [21-23]. These two proteins lack signal sequences in their primary structures, and cannot be released through the classical endoplasmic reticulum-Golgi pathway. Their nonclassical export involves copper-dependent association with a small calcium-binding protein, S100A13.

Tetrathiomolybdate (TM), which forms a stable tripartite complex with copper and protein, is a copper-lowering agent that has been evaluated extensively in the treatment of Wilson's disease [24]. TM treatment decreases serum copper levels and attenuates angiogenesis and tumor growth in animal tumor models [25-27]. The hypothesis underlying this approach is that one or more copper-containing or copper-binding angiogenic proteins (e.g. VEGF, FGF-1, FGF-2, angiogenin, angiotropin, or others) require higher levels of copper to be active than are required for basic cellular needs [28]. In fact, the antitumor activity of TM was evaluated in patients with advanced kidney cancer in a phase II trial [29].

Alternatively, the *in vivo *effects of TM may be explained by its ability to block the release of FGF-1 and IL-1α, both known as potent proangiogenic and proinflammatory polypeptides. Indeed, the inhibition of restenosis by TM in the model of damaged rat carotid artery was accompanied by the downregulation of FGF-1 and IL-1α levels in the vessel wall [22]. Additionally, copper is known to play an important role in the development and maintenance of the immune system [30]. Some reports revealed that the possibility of inhibiting both fibrotic response and inflammatory response by copper chelation is due to the suppression of transforming growth factor beta and TNF-α production [31,32].

The serum copper level in RA patients has been reported to be high [33], and the IL-1β and TNF-α serum content might correlate with the serum copper level in RA patients [34]. In addition, D-penicillamine (another anticopper agent) has been used as the therapy for RA for many years. A previous study suggested that D-penicillamine might regress rheumatoid synovial hyperplasia via Fas-mediated apoptosis, but the mechanism of the effect of D-penicillamine is still unknown [35]. In animal studies, TM is a more fast-acting, more potent, copper chelating agent than D-penicillamine [36]. We hypothesized that the anticopper drug TM can be useful for the treatment of RA through inhibition of proangiogenic and proinflammatory cytokines. We examined whether TM has the potency to suppress chronic inflammation, pannus formation, and angiogenesis in the course of adjuvant-induced arthritis (AIA) in female Lewis rats. We also examined whether copper chelation with TM reduces the production of VEGF in serum and synovium of AIA rats and suppresses inflammatory cachexia in AIA rats.

## Materials and methods

### AIA in rats

Eight-week-old female Lewis rats were obtained from Charles River Japan (Yokohama, Japan). Complete Freund's adjuvant was prepared by suspending heat-killed *Mycobacterium butyricum *(Difco Laboratories, Detroit, MI, USA) in liquid paraffin (Merck & Co., Whitehouse Station, NJ, USA) (a kind gift from Nippon Shinyaku, Kyoto, Japan) at 12 mg/ml. Complete Freund's adjuvant-induced arthritis was stimulated by injection of 50 μl Complete Freund's adjuvant emulsion intradermally at the base of the tail, as described previously [37-39]. This experimental procedure was designed and carried out according to the institutional rules and regulations of the animal research of Kyoto Prefectural University of Medicine.

### Administration of TM

TM treatment commenced 2 weeks before immunization; TM (10 mg/kg) was given in 3 ml water once daily by means of intragastric gavage until 10 days after the onset of arthritis. Deionized water was given to control rats. The animals were housed four to a cage at 21°C in a 12-hour light/dark cycle. The rats were killed on day 17 after immunization under anesthesia with sodium pentobarbital.

### Evaluation of arthritis

From day 7 after immunization (onset of arthritis), rats were examined every 2 days for three clinical parameters: paw volume, arthritis score, and body weight. The footpad volume was measured with a water replacement plethysmometer (Unicom Japan, Tokyo, Japan). For clinical evaluation of AIA, the mid-forepaw, the wrist, the joints of the finger, the midfoot, the ankle, and the joints of the digits were scored on a 0–4 scale: 0, normal; 1, minimal swelling; 2, medium swelling; 3, severe swelling; 4, severe and nonweight-bearing arthritis. Each limb was graded, resulting in a maximal clinical score of 48 per animal.

### Copper status

In the course of TM therapy, the copper status cannot be assessed by direct measurement of serum copper. The accumulation of a tripartite complex of TM, copper, and albumin turns over slowly. The serum copper is therefore increased even though the availability of copper is decreased. Serum ceruloplasmin is a good surrogate marker of copper status because the liver secretes this copper-containing protein into the blood at a rate dependent on copper availability [40].

We monitored the copper status by assaying serum ceruloplasmin in blood from the tail vein. The ceruloplasmin measurements were made by nephelometry (differential light scattering from a colored or turbid case solution with respect to a control solution) using an automated system and reagents available commercially (Dade Behring Inc., Deerfield, IL, USA) when TM-treated rats were immunized.

### Histological examination

After euthanasia on day 17, the hindpaws were amputated above the knee joint and were fixed in 7.4% formaldehyde solution. The paws were then decalcified, embedded in paraffin, and sectioned in a mid-sagittal plane. The sections of articulation of the tarsal joints were stained with hematoxylin and eosin, and were examined microscopically. We also performed the hematoxylin and eosin staining of tissue specimens of the liver and the kidney.

Two blinded observers evaluated cartilage and bone destruction by pannus formation, mononuclear cell infiltration, and vascularity in synovial tissues in each preparation on two separate occasions, using the following scoring system [41]: mononuclear cell infiltration (0, no infiltration; 1, mild infiltration; 2, moderate infiltration; 3, severe infiltration); cartilage and bone destruction by pannus formation (0, no change; 1, mild change [pannus invasion within cartilage]; 2, moderate change [pannus invasion into cartilage/subchondral bone]; 3, severe change [pannus invasion into the subchondral bone]); and vascularity (0, almost no blood vessels; 1, a few blood vessels; 2, some blood vessels; 3, many blood vessels).

### Immunostaining

VEGF, CD11b, and von Willebrand factor (vWF) chain antigens were detected by the use of saturating amounts of antibodies against VEGF, CD11b, and vWF in combinations with immunoperoxidase staining with a Vectastain ABC kit (Vector Laboratories, Burlingame, CA, USA) according to the manufacturer's protocol [14]. The sections were deparaffinized with xylene and graded ethanol and were immersed in 0.3% peroxidase in 90% methanol for 45 min in order to exhaust endogenous peroxidase. They were preincubated with 0.2% bovine serum albumin in PBS for 20 min and with diluted normal horse serum (1:66.7), or normal goat serum (1:66.7) for 30 min followed by incubation with 5 μg/ml anti-VEGF monoclonal IgG antibody (sc-7269: Santa Cruz Biotechnology, Santa Cruz, CA, USA), 10 μg/ml anti-CD11b monoclonal antibody (Serotec Ltd, Kidlington, UK), 10 μg/ml anti monoclonal vWF antibody (Sigma, St Louis, MO, USA), 5 μg/ml purified rabbit IgG (Vector Laboratories), or 5 μg/ml purified mouse IgG (Vector Laboratories) for 16 hours in a humid chamber at 4°C. After washing with PBS, the sections were incubated with biotinylated horse anti-mouse IgG (Vector Laboratories) or goat anti-rabbit IgG (Vector Laboratories) for 30 min. Then, after again washing with PBS, the sections were incubated with avidin and further incubated with biotinylated horseradish peroxidase complex, each for 45 min. Finally, the sections were washed with PBS for 10 min and developed by immersing in a solution of 0.05% (w/v) 3,3'-diaminobenzidine tetrahydrochloride (Sigma Chemical, St Louis, MO, USA), and 0.01% hydrogen peroxide in 0.05 M Tris (pH 7.4) for 2 min. The sections were then counterstained with hematoxylin for 2 min, dehydrated with graded ethanol and xylene for 1 min, respectively, and finally coverslips were mounted.

For both tissue specimens from TM-treated rats and control rats, the extent and intensity of staining with anti-VEGF antibody in synovial lining cells, macrophages, endothelial cells, and fibroblasts were graded on a scale of 0–3+ by two blinded observers on two separate occasions using coded slides as previously described [37]. A 3+ grade implies maximally intense staining, whereas 0 implies no staining.

CD11b immunostaining in monocytes was used to evaluate mononuclear cell infiltration for each of tissue specimens. vWF immunostaining in endothelial cells was used to evaluate vascularity for each of the tissue specimens.

### Measurement of body weight of AIA rats

From day 7 after immunization (onset of arthritis), the body weight of AIA rats was examined every 2 days. We also measured body weights of rats at the first administration of TM or of deionized water. At this initial time point, the differences between the two groups were not significant. We also measured body weights of 10-week-old female Lewis rats (*n *= 10), which were not immunized, for a period of 14 days as a negative control.

### Measurement of VEGF production in rats

When rats were sacrificed, the serum was collected. We measured the concentrations of VEGF in the serum using the VEGF ELISA kit (Biosource International, Camarillo, CA, USA).

### Statistical analysis

Two-way analysis of variance was used to test the statistical significance of differences between a TM-treated group and a control group for the analysis of hindlimb paw volume, body weight, and clinical score of arthritis. The Mann–Whitney U test to compare nonparametric data for statistical significance was applied for the analysis of histological scores. A nonpaired *t *test was used for the analysis of the serum concentration of VEGF. The Spearman coefficient of correlation was used to examine the correlation between the extent of pannus formation and the VEGF level in the serum.

## Results

### Oral administration of TTM attenuates AIA

**Figure 1:**
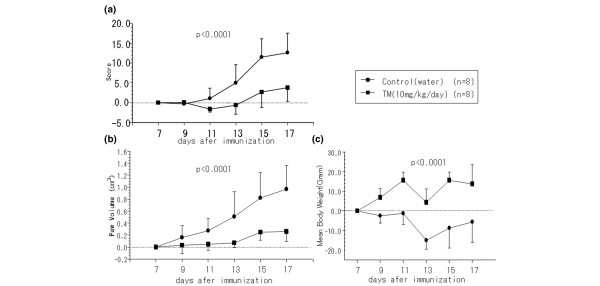
Suppression of clinical arthritis by tetrathiomolybdate (TM) treatment in adjuvant-induced arthritis rats. TM was orally administered daily to female Lewis rats, from 2 weeks before immunization to day 17. TM-treated rats, 10 mg/kg/day (*n *= 8); control rats (*n *= 8). **(a) **Arthritis score, **(b) **paw volume, and **(c) **mean body weight were assessed every second day after onset of arthritis.

**Figure 2:**
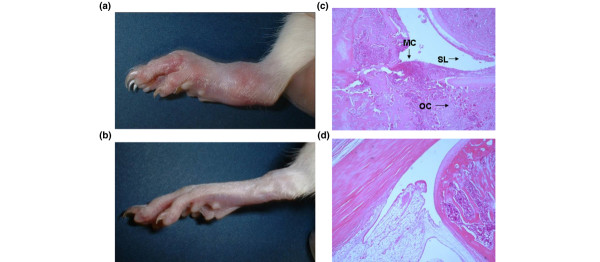
Morphological features and histopathological aspects of the hindlimb in AIA rats. **(a) **Control rats and **(b) **rats treated with tetrathiomolybdate (TM). Joint swelling, redness, and edema of the foot in AIA was clearly reduced with TM administration at day 17 after immunization. Histopathological studies using hematoxylin and eosin staining of the foot joint also revealed **(c) **a marked decrease of synovial inflammatory cell infiltrate and synovial lining hyperplasia compared with **(d) **control rats. (c), (d) Original magnification × 40. AIA, adjuvant-induced arthritis; MC, monocyte; SL, synovial lining cell; OC, osteoclast.

To explore the effect of TM on AIA rats, TM (10 mg/kg) was administered daily starting from 14 days before immunization. Inflammatory polyarthritis was induced in all nontreated immunized rats and it occurred on day 8 after immunization (day 7 in Materials and methods). TM administration delayed the onset and suppressed severity of clinical arthritis comparative to control rats fed with deionized water, as demonstrated by both the paw volume (Figs 1a and 2) (*P *< 0.0001) and the arthritis score (Fig. 1b) (*P *< 0.0001). Especially significant TM effects were observed at days 11–17 after immunization. These data suggest that oral TM administration inhibits the onset of and reduces the severity of arthritis in AIA rats. The body weight of TM-treated rats was significantly increased compared with control AIA rats (Fig. 1c) (*P *< 0.0001). We also measured the body weights of 10-week-old female Lewis rats (*n *= 10), which were not immunized, for a period of 14 days. The results show that the body weight of control AIA rats was significantly decreased compared with that of nonimmunized normal rats, and the body weight gain of TM-treated AIA rats was almost similar to that of nonimmunized normal rats (data not shown). These data suggest that oral TM administration prevents inflammatory body weight loss in AIA rats.

### Histological effects of TM in the foot joint of AIA rats

**Figure 3:**
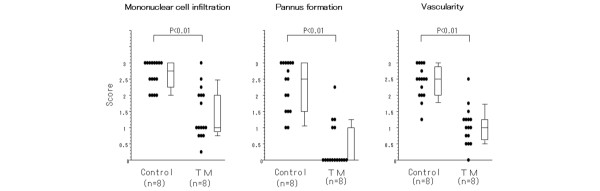
Histopathological scores of the hindlimb in AIA rats fed with TM or deionized water. Mononuclear cell infiltration, pannus invasion into the cartilage and bone, and vascularity were measured by microscopic examination scores of the sections on two separate occasions (see Histological examination). We measured the scores of 16 hindlimbs (both hindlimbs of each rat). AIA, adjuvant-induced arthritis; TM, tetrathiomolybdate.

At day 17, histological study of foot joints (shown in Fig. 2) in TM-treated rats revealed that the infiltration of mononuclear cells, the formation of pannus in synovial tissues, and the extent of vascularity were significantly decreased compared with control rats (*P *< 0.01; Fig. 3) (data of immunostaining of CD11b and vWF not shown). These data suggest that TM exhibits anti-inflammatory and anti-angiogenic effects, and inhibits the growth of synoviocytes in AIA.

### VEGF expression in synovial tissues in AIA rats

**Figure 4:**
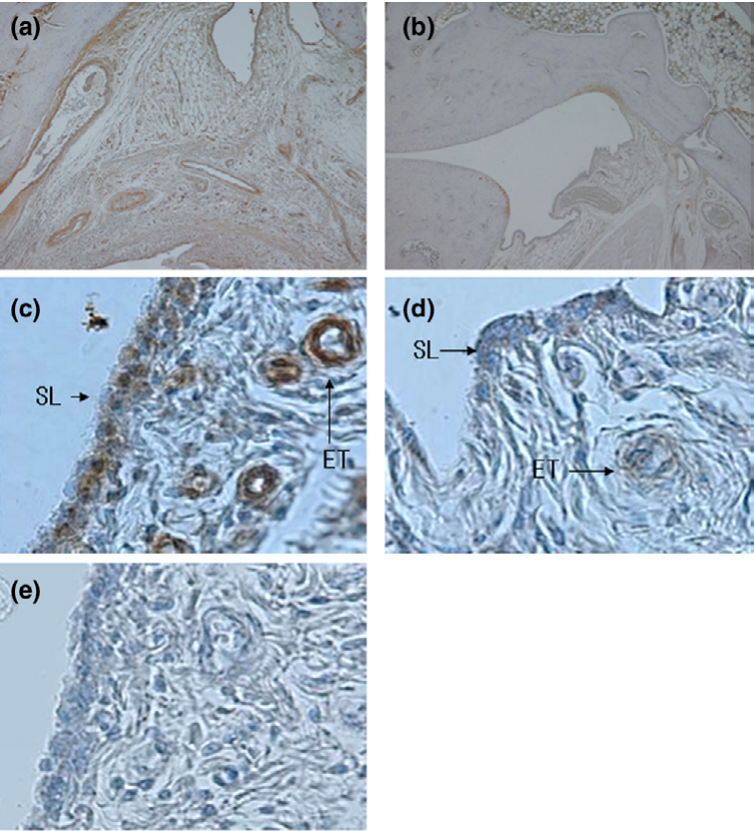
Immnunostaining for VEGF in synovial tissue in AIA rats. **(a)**,**(c)**, **(e) **AIA rats treated with deionized water and **(b)**,**(d) **tetrathiomolybdate (TM). Immunohistochemical staining was performed with the Vecto Stain avidin–biotin peroxidase complex kit (Vector Laboratories, Burlingame, CA, USA). Synovial tissues sections were stained with (a)–(d) mouse anti-VEGF monoclonal IgG antibodies (1:200 dilution, in PBS; Santa Cruz Biotechnology, Santa Cruz California, USA) and (e) a normal mouse IgG (1:200 dilution, in PBS). Positive immunostaining was indicated by brownish deposits. The counterstain was an aqueous solution of hematoxylin. (a), (b) Original magnification × 40; (c)–(e) original magnification × 400. AIA, adjuvant-induced arthritis; ET, endothelial cell; SL, synovial lining cell.

Immunohistochemistry was used to examine the expression and localization of VEGF in AIA rats (Fig. 4). We found markedly enhanced expression of VEGF in endothelial cells (immunohistochemical score 2.1 ± 0.5), and found moderate expression in fibroblasts (1.7 ± 0.6) and macrophages (1.6 ± 0.9) of immunized rats. In control nonimmunized rats (*n *= 6), the expression of VEGF was 1.1 ± 0.5 in endothelial cells, was 0.6 ± 0.3 in fibroblasts, and was 0.1 ± 0.0 in macrophages. In TM-treated immunized rats, the localization of VEGF in synovial tissues was similar to that in control immunized rats. However, the immunohistochemical score of VEGF in TM-treated immunized rats in endothelial cells (1.1 ± 0.5, *P *< 0.01), in macrophages (0.7 ± 0.7, *P *< 0.01), and in fibroblasts (1.2 ± 0.5, *P *< 0.05) was significantly lower than in control immunized rats. Control immunostaining with normal mouse serum was completely negative in all animals.

### TM effect on VEGF production in AIA rats

**Figure 5:**
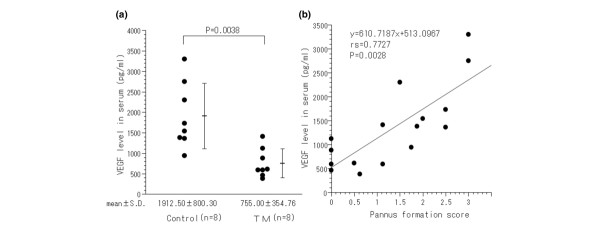
VEGF levels in the serum are correlated with the extent of arthritis in rats. **(a) **VEGF levels in the serum are higher in control rats than in tetrathiomolybdate (TM)-treated rats (* *P *< 0.01). VEGF was analyzed by ELISA, as described in Materials and methods. Data represent the mean ± standard deviation. **(b) **A significant positive relationship between VEGF levels in the serum and the severity of pannus formation in hindlimbs of adjuvant-induced arthritis rats, which includes both the control group and the TM-treated group. VEGF, vascular endothelial growth factor.

To examine the effect of TM upon VEGF production, we measured the VEGF level in the serum of TM-treated rats and control AIA rats (Fig. 5a). The production of VEGF in the serum was significantly suppressed by TM treatment (755.0 ± 354.7 pg/ml versus 1912.5 ± 800.3 pg/ml in TM-untreated animals, *P *= 0.0038). Interestingly, the extent of pannus formation significantly correlated with the production of VEGF in the serum (Fig. 5b).

### Side effects of oral administration of TM

There was no mortality in TM-treated rats. The liver and kidney histological examination in TM-treated rats did not show any abnormalities, and we could not detect any pathological changes when comparing them with TM-untreated rats.

## Discussion

We demonstrated that TM therapy has a strong protective effect against progression of AIA in rats. Usually AIA is accompanied by weight loss because of inflammatory response, but TM prevents this. TM also significantly reduces the level of VEGF in the serum and inhibits the expression of VEGF in synovial tissues, especially in endothelial cells and macrophages. Joint and bone destruction due to arthritis are markedly suppressed by TM, and the extent of bone destruction is significantly correlated with the production of VEGF in the serum.

An increase of serum copper and ceruloplasmin concentrations has been demonstrated in RA patients [34,42]. In RA these parameters are measures of disease, and they do not depend on dietary factors [43]. Acute or chronic inflammatory processes cause an accumulation of zinc and copper in many organs, particularly in the inflamed areas [44]. Additionally, a number of biologically active extracellular polypeptides, including cytokines and angiogenic factors, which participate in the pathogenesis and development of inflammatory processes, are known to be involved in trace metal metabolism. Copper plays an important role in development and maintenance of the immune system [30]. IL-1α and FGF-1 are Cu^2+^-binding proteins. The stress-induced IL-1α and FGF-1 release pathways in murine NIH 3T3 cells and human U937 cells are sensitive to TM treatment [45]. The presence of copper in cell cultures is essential for T-cell proliferation induced by macrophages or by macrophage-mediated cytokines [30]. A recent study revealed that IL-1β and TNF-α levels significantly correlate with serum copper concentrations [34]. In our study, *in vivo*, copper chelation with TM strongly repressed acute inflammation and onset of AIA through inhibition of mononuclear cell infiltration, and pannus formation.

TM has been shown to be a potent anti-angiogenic and anti-metastatic compound in tumors. In a phase II clinical trial for advanced renal cell carcinoma, patients rendered copper deficient with TM therapy had a significantly decreased serum content of proangiogenic mediators, VEGF, FGF-2, IL-6, and IL-8 [29]. In RA patients, proangiogenic factors such as VEGF play an important role in pathological angiogenesis, and the other factors such as IL-6 and IL-8 are considered to have additional effects on its development. VEGF is also a key player in pannus development, acting through the VEGF receptor I signaling pathway. The blockade of the VEGF receptor I suppresses joint destruction in the K/BxN model of RA [46], and serum concentrations of VEGF are elevated in RA patients and correlate with disease activity [47]. In our study, the onset of AIA in rats is delayed, and its severity is suppressed by TM administration through the inhibition of pannus formation and angiogenesis. The anti-arthritic effect of TM might therefore result from the inhibition of VEGF production by synovial tissues. We also examined the efficacy of TM administration starting from day 7 after immunization (the onset of the disease) for AIA in rats; in this case, TM had only a mild anti-arthritic effect. Apparently, to efficiently attenuate arthritis, copper depletion needs to be achieved by the moment of its onset.

TM was well tolerated in patients with advanced kidney cancer in a phase II trial, with dose reductions most commonly occurring for grade 3–4 granulocytopenia of short duration not associated with febrile episodes [29]. The principal features observed in severe copper deficiency are anemia, neutropenia, and osteoporosis. TM was remarkably nontoxic when ceruloplasmin was lowered to 10–20% of baseline levels for up to 17 months of treatment, and the only drug-related toxicity observed was mild anemia, which was easily reversible with adjustment of the TM dose to bring the ceruloplasmin level to the desired target [40]. But various side effects may occur when ceruloplasmin is reduced below 5 mg/dl, such as bone marrow suppression, diarrhea, and arrhythmia. We assayed the serum ceruloplasmin level as a surrogate marker of copper status, and kept it in a range between 5 and 10 mg/dl in TM-treated rats. As the histological examination of the liver and kidney demonstrated, no significant adverse effects were observed. This means that the extent of copper chelation in this study was sufficient and not excessive.

We have demonstrated that TM administration prevents cachexia, which is associated with RA. It is known that AIA in rats is a useful model of inflammatory cachexia that mimics the human pathophysiology in important ways, and is consistent with cytokine-driven cachexia in chronic inflammatory arthritis [48]. We found that the body weight of control AIA rats was significantly decreased compared with that of nonimmunized normal rats, and the body weight gain of TM-treated AIA rats was almost similar to that of nonimmunized normal rats. Rheumatoid cachexia is characterized by altered energy and protein metabolism (reduced total energy expenditure, increased resting energy expenditure, and increased whole-body protein catabolism) and increased inflammatory cytokine production [15].

Leptin is a peptide hormone-regulating body weight, and it exhibits a variety of other effects including the regulation of the endocrine system, reproduction, and immunity [49,50]. The severity of antigen-induced arthritis is decreased in leptin-deficient ob/ob mice [49]. Furthermore, serum leptin levels in patients with RA are significantly higher than those in control patients, and leptin stimulates proinflammatory cytokine production in monocytes and macrophages *in vitro *[51]. Moreover, it is reported that TM therapy resulted in significantly reduced body-weight loss caused by bleomycin-induced pulmonary fibrosis in mice [31].

These findings suggest that copper chelation by TM may not only suppress joint destruction, but also may influence energy and protein metabolism in the course of RA through the upregulation of the adipocytokine leptin.

## Conclusion

TM therapy had a strong protective effect against progression of adjuvant arthritis in rats and inflammatory cachexia with minimal adverse effects. TM also significantly reduced the content of VEGF in the serum and synovial tissues. Joint and bone destruction due to arthritis was markedly suppressed with TM. The extent of bone destruction was correlated with the production of VEGF in the serum.

TM is a potential therapeutic candidate for the treatment of angiogenic and inflammatory diseases in which the serum copper and VEGF levels are elevated. Additionally, the efficacy of TM against inflammatory cachexia may be useful for improving the well-being of RA patents.

## Abbreviations

AIA = adjuvant-induced arthritis; ELISA = enzyme-linked immunosorbent assay; FGF = fibroblast growth factor; IL = interleukin; PBS = phosphate-buffered saline; RA = rheumatoid arthritis; TM = tetrathiomolybdate; TNF-α = tumor necrosis factor alpha; VEGF = vascular endothelial growth factor; vWF = von Willebrand factor.

## Competing interests

The author(s) declare that they have no competing interests.

## Authors' contributions

AO conceived of the study, participated in its design, and performed all the experiments. YK conceived of the study, participated in the design of and the coordination of the study, and participated in the interpretation of the results. IP and HS participated in the design of the animal study. TY, YT, and RY participated in the immunohistochemistry and performed the interpretation of the results. MK and HI performed the animal study. MW, MK, and MY participated in the immunoassay. All authors read and approved the final manuscript.
